# Rubiadin, as a key metabolite of the Bushen Huoxue formula, promotes apoptosis of endometrial stromal cells and improves intrauterine adhesions by activating the AMPK/p53/p21 pathway

**DOI:** 10.3389/fphar.2026.1732284

**Published:** 2026-04-24

**Authors:** Cong Shen, Chenyang Sha, Mengqi Yang, Yi Lin, Juanjuan Ma, Dongyi Shen, Baohua Zhang, Cong Qi, Qinhua Zhang, Xiaole Zhang

**Affiliations:** 1 Department of Obstetrics and Gynecology, Shuguang Hospital Affiliated to Shanghai University of Traditional Chinese Medicine, Shanghai, China; 2 Department of Obstetrics and Gynecology, Punan Branch of Renji Hospital, Shanghai Jiaotong University School of Medicine, Shanghai, China

**Keywords:** AMPK-p53-p21 pathway, apoptosis of endometrial stromal cells, Bushen Huoxue formula, intrauterine adhesions, Rubiadin

## Abstract

**Background:**

*The Bushen Huoxue formula (BSHX)* is a classic traditional Chinese medicine prescription for treating gynecological disorders. However, its specific metabolites and mechanisms against IUA remain to be elucidated.

**Aim:**

This study aimed to identify the key metabolite of BSHX and to investigate its therapeutic effects and underlying mechanism against IUA, both *in vitro* and *in vivo*.

**Methods:**

Network pharmacology and molecular dynamics simulation were utilized to screen the metabolites of BSHX. *In vitro*, cells were treated with different concentrations of Rubiadin with or without the AMPK inhibitor Dorsomorphin. Cell viability, apoptosis, migration, invasion, and pathway activity were assessed. *In vivo*, the IUA rat model was treated with Rubiadin and Dorsomorphin, and histopathology, fibrosis, inflammation, pregnancy outcomes, and associated signaling pathways were evaluated.

**Results:**

In IUA clinical tissues, the AMPK/p53/p21 pathway was significantly inhibited. Rubiadin dose-dependently activated the AMPK/p53/p21 pathway, promoted endometrial stromal cell apoptosis, and inhibited cell migration, invasion, and collagen synthesis *in vitro*. These effects were reversed by Dorsomorphin. In IUA rats, Rubiadin treatment activated the AMPK/p53/p21 pathway, reduced endometrial fibrosis and inflammation, and significantly improved pregnancy rates. The AMPK inhibitor attenuated these therapeutic benefits.

**Conclusion:**

Rubiadin, a key metabolite of the Bushen Huoxue formula, ameliorates intrauterine adhesions by activating the AMPK/p53/p21 pathway, which promotes the apoptosis of endometrial stromal cells and restores endometrial function. This study provides a pharmacological basis for using Rubiadin as a potential therapeutic agent for IUA.

## Highlights

- Rubiadin from Bushen Huoxue formula activates the AMPK/p53/p21 pathway in endometrial stromal cells.
- Rubiadin promotes apoptosis and inhibits fibrosis in a dose-dependent manner *in vitro*.
- Rubiadin ameliorates intrauterine adhesions and improves pregnancy rates in a rat IUA model.
- The therapeutic effects of Rubiadin are mediated through AMPK-dependent mechanisms.

## 1 Background

Intrauterine adhesion (IUA), also known as Asherman syndrome, was first defined by Joseph Asherman in 1948. The development of IUA is a significant potential fertility complication arising from hysteroscopic surgery. It is characterized by the formation of filmy or dense fibrous adhesions within the uterine cavity, resulting in the adherence of the uterine walls to each other (Healy M. W. et al., 2016; Asherman J. G., 1948). The incidence of IUA has been increasing over the past few decades, with main clinical manifestations including menstrual abnormalities (such as hypomenorrhea), secondary infertility, recurrent miscarriage, and pelvic pain. Among these, secondary infertility has a particularly severe impact on patients’ quality of life (Healy M. W. et al., 2016; Pongpattanawut et al., 2016; Di Guardo et al., 2020; Deans and Abbott, 2010). Currently, the standard treatment for moderate to severe IUA is transcervical resection of adhesion (TCRA) (Johary et al., 2014), which aims to reshape the uterine cavity volume and morphology to enhance fertility potential. However, the postoperative recurrence rate of adhesions is high, reaching 62% or more in patients with severe IUA (Capella-Allouc S. et al., 1999). Estrogen therapy is commonly used to prevent postoperative adhesion recurrence, but its effect is limited when used alone for moderate to severe IUA, and the reproductive prognosis of patients remains unsatisfactory (Xiao S. et al., 2014). Currently, treatment options for intrauterine adhesions are very limited due to the lack of effective methods to promote regeneration after severe endometrial injury (Jiang X. et al., 2021). The core pathological mechanism of IUA is closely related to the inhibition of endometrial stromal cell apoptosis and the imbalance of extracellular matrix metabolism (Johary J. et al., 2014; Liao L. et al., 2024). However, the specific regulatory network has not been fully elucidated. Therefore, exploring more effective strategies to improve the reproductive outcomes of IUA patients is particularly crucial.

The kidney-tonifying and blood-activating therapy is an important therapeutic method in traditional Chinese medicine (TCM), widely used in the treatment of various clinical diseases (Lin X. et al., 2015). *The Bushen Huoxue formula* is a classic prescription in TCM for treating gynecological blood stasis syndrome, composed of *Astragalus membranaceus*, *Angelica sinensis, Ligusticum chuanxiong*, etc., with the effects of tonifying the kidney, benefiting qi, activating blood circulation, and resolving blood stasis. This formula is not only believed to significantly alleviate tissue fibrosis and suppuration (Lin Y. et al., 2023), but also applicable to the treatment of inflammatory cytokines (Lv H. et al., 2023). In addition, its roles in protecting ovarian function and improving pregnancy rates are particularly prominent (Ma et al., 2023; McCallum et al., 2018). TCM has unique advantages in endometrial repair (Meng W. H. et al., 2024). Modern pharmacological studies have confirmed that its metabolites can improve endometrial repair through anti-inflammatory effects (Niu H. et al., 2021), promoting endometrial proliferation (Peng W. et al., 2024), regulating cell proliferation and apoptosis (Pongpattanawut C. et al., 2016), and other mechanisms. The Bushen Huoxue formula is a metabolite formulation of traditional Chinese medicine that integrates kidney tonification and blood activation functions. It has demonstrated significant efficacy in improving female reproductive health conditions such as menstrual disorders, premature ovarian insufficiency, and infertility (Song Y. T. et al., 2021). Recent studies have shown that the Bushen Huoxue formula may inhibit the fibrotic process through the ADAM17/Notch signaling pathway, indicating that it is one of the potential methods for treating IUA (Tang S. S. et al., 2025). However, its specific role and molecular targets in clinical samples of intrauterine adhesion tissues remain unclear. Through this network pharmacology screening, Rubiadin was screened out as a promising candidate owing to its high binding affinity with the core target AMPK. Rubiadin, a 1,3-dihydroxy-2-methyl anthraquinone primarily derived from *Rubia cordifolia* Linn (Rubiaceae), demonstrates a broad spectrum of pharmacological properties, including anticancer, anti-osteoporotic, hepatoprotective, neuroprotective, anti-inflammatory, antidiabetic, antioxidant, antimicrobial, antimalarial, antifungal, and antiviral activities (Watroly M. N. et al., 2021).

The AMPK-p53-p21 pathway is a core pathway for cellular energy sensing and apoptosis regulation: activated AMPK can phosphorylate p53, promote p21 expression, thereby inducing cell cycle arrest and apoptosis, and simultaneously inhibiting extracellular matrix synthesis (Wang and You, 2016). Existing studies have shown that C1q/tumor necrosis factor-related protein-6 (CTRP6) exerts an anti-fibrotic effect by activating the AMPK signaling pathway, thereby ameliorating intrauterine adhesions (Xu R. et al., 2024). In addition to this, AMPK plays a crucial role in postpartum endometrial regeneration and tissue remodeling accompanied by decidualization (Yan S. et al., 2024). However, there is currently no direct evidence whether the AMPK-p53-p21 pathway is involved in the pathological process of intrauterine adhesions by regulating endometrial stromal cell apoptosis, and whether it can be used as a therapeutic target.

**FIGURE 1:**
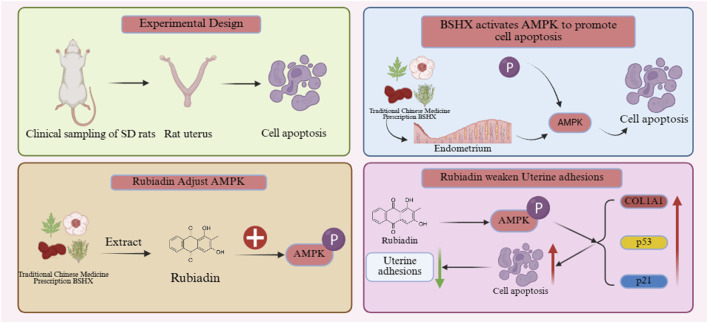
Experimental protocol. This figure illustrates the mechanism by which the traditional Chinese medicine prescription BSIX and its metabolite Rubiadin affect uterine cell apoptosis and adhesion through regulating the AMPK pathway.

In summary, this study focuses on patients from 2022 to 2024 as research subjects, combined with a rat model of IUA, to explore the effects of metabolites of the Bushen Huoxue formula on the proliferation and apoptosis of human endometrial stromal cells and its mechanism, aiming to provide a new theoretical basis and potential drug targets for the prevention and treatment of intrauterine adhesions (Figure 1).

## 2 Materials and methods

### 2.1 Preparation and composition identification of BSHX

The Bushen Huoxue formula (BSHX) is a classical multi-botanical drugs prescription composed of Tusizi 15g, Dihuang 12g, Bajitian 15g, Huangqi 30g, Danggui 15g, Niuxi 18g, Chuanxiong9g, Gancao 6 g (Table 1). All botanical drugs were obtained from a certified supplier (Shanghai Kangqiao Traditional Chinese Medicine Co., Ltd., China) and identified by a licensed taxonomist (Dr. Liu, Shanghai University of Traditional Chinese Medicine). Voucher specimens (BSHX-2023–001 to BSHX-2023–008) are deposited in the Herbarium of Shuguang Hospital. Species names were verified using the Kew Medicinal Plant Names Services (MPNS, http://mpns.kew.org) and Plants of the World Online (http://www.plantsoftheworldonline.org). All plant materials comply with the Nagoya Protocol and relevant national regulations.

**TABLE 1:** Traditional botanical drug remedies of BSHX.

Family	TCM herds	Original plants	Part(s) of plant used
Convolvulaceae	Tusizi	Cuscuta chinensis Lam	Dried ripe seed
Rubiaceae	Bajitian	Gynochthodes officinalis (F.C.How) Razafim. and B.Bremer	Dried root
Fabaceae	Huangqi	Astragalus mongholicus Bunge	Dried root
Apiaceae	Danggui	Angelica dahurica (Hoffm.) Benth. and Hook.f. ex Franch. and Sav	Dried root
Orobanchaceae	Dihuang	Rehmannia glutinosa (Gaertn.) Libosch. ex DC.	Prepared root tuber
Apiaceae	Chuanxiong	Conioselinum anthriscoides ‘Chuanxiong'	Dried rhizome
Fabaceae	Gancao	Glycyrrhiza glabra L. ,Glycyrrhiza uralensis Fisch. ex DC. and Glycyrrhiza inflata Batalin	Honey-fired root and rhizome
Amaranthaceae	Niuxi	Achyranthes bidentata Blume	Dried root

The formula was prepared according to the standard decoction protocol described in the Pharmacopoeia of the People’s Republic of China (2020 Edition). Briefly, dried plant materials were mixed in the prescribed ratio, soaked in distilled water (1:10 w/v), and extracted twice by reflux (1 h each). The combined filtrates were concentrated under reduced pressure and lyophilized to obtain the crude extract (yield: 18.7%).

Chemical profiling was performed using ultra-high-performance liquid chromatography coupled with quadrupole time-of-flight mass spectrometry (UHPLC-QTOF-MS; Agilent 1290/6545). A Dionex Ultimate 3000 high-performance liquid chromatography (HPLC) system was employed for separation. The mobile phase consisted of a gradient elution of 0.1% formic acid in methanol, delivered at a flow rate of 0.3 mL/min. Chromatographic separation was performed on a column maintained at 40 °C, with an injection volume of 3 μL. Mass spectrometric analysis was conducted using a Q Exactive quadrupole–electrostatic field orbitrap high-resolution mass spectrometer equipped with a heated electrospray ionization (H-ESI) source. The auxiliary gas flow was set to 13 arbitrary units (arb), while the auxiliary heater and ion transfer tube temperatures were maintained at 300 °C and 320 °C, respectively. The automatic gain control (AGC) target was 1 × 10^6^, and the RF level (S-lens voltage) was set to 50. For negative-ion mode, the sheath gas (N_2_) flow was 35 arb and the spray voltage was −2.5 kV; for positive-ion mode, the sheath gas (N_2_) flow remained at 35 arb, and the spray voltage was +3.5 kV. Data acquisition was performed in both positive- and negative-ion data-dependent MS/MS (dd-MS^2^) mode (Full MS/dd-MS^2^), comprising: (i) a full-scan MS survey (resolution: 70,000 FWHM at *m/z* 200) over the range *m/z* 100–1500; and (ii) subsequent data-dependent MS/MS scans (resolution: 17,500 FWHM at *m/z* 200) triggered from the top 10 most abundant precursor ions per cycle. Collision energy was stepped across three levels: 10, 20, and 40 eV. Raw data were processed using ProteoWizard and an in-house R package integrated into the Biotree TCM/BT-HERB database (Supplementary Tables S3, S4; Supplementary Figure S6).

### 2.2 Metabolites and reagents

Rubiadin (purity ≥98%, CAS No. 117–02-2 and Dorsomorphin (Metabolite C, CAS No. 866405–64-3; Batch No. HY-13418) were purchased from MedChemExpress (MCE, USA). All antibodies, assay kits, and cell culture reagents were obtained from commercial sources as listed in Supplementary Material S1, S2. Human primary endometrial stromal cells (hESCs) were purchased from Otwo Biotech (Cat# HTX-2022, Lot# 220315; donor age 28–35 years, passage 3–5; mycoplasma-free certificate provided). Cells were cultured in DMEM/F-12 medium supplemented with 10% fetal bovine serum (FBS), 100 U/mL penicillin, and 100 μg/mL streptomycin at 37 °C with 5% CO_2_.

### 2.3 Clinical sample collection and ethics

This study was approved by the Ethics Committee of Shuguang Hospital (Approval No. 2024-1053-296-01) and conducted in accordance with the Declaration of Helsinki. All participants provided written informed consent. Endometrial tissues were collected from patients undergoing hysteroscopic surgery: three intrauterine adhesion (IUA) samples (from adhesiolysis) and three normal endometrial samples (from benign gynecological surgery). Tissues were either snap-frozen in liquid nitrogen or fixed in 4% paraformaldehyde for subsequent analysis.

### 2.4 Cell experiment grouping

Endometrial stromal cells were purchased from Otwo Biotech. The dose-dependent experiment included a Control group (treated with an equal volume of solvent), a Rubiadin-L group (with a final concentration of 20 μM), a Rubiadin-M group (with a final concentration of 40 μM), and a Rubiadin-H group (with a final concentration of 50 μM); meanwhile, the pathway mechanism experiment consisted of a Control group, a Rubiadin-H group, and a Rubiadin-H + Dorsomorphin group (Dorsomorphin at a final concentration of 1 μM) (Chee and Zamakshshari, 2022; Zhang et al., 2025).

Cells were cultured in medium containing fetal bovine serum, 100 U/mL penicillin, and 100 μg/mL streptomycin, in a 37 °C, 5% CO_2_ incubator.

### 2.5 Cytoplasmic plasmid construct and transfection

The AMPK-specific interference sequence (shAMPK) and the negative control sequence (shNC) were transfected into human endometrial stromal cells (hESCs) using Lipofectamine 3000. After transfection, qPCR and Western blot techniques were used to verify the gene knockdown efficiency. Relevant sequence information and verification data are shown in Supplementary Table S2 (refer to sequence NM_006251).

One day before transfection, seed cells in the logarithmic growth phase into a 6-well plate at an appropriate density, adding 2 mL of complete medium containing serum but no antibiotics to each well. Incubate in a 37 °C, 5% CO_2_ incubator so that the cell confluency reaches 70%–90% at the time of transfection. Adjust the concentration of plasmid DNA to 1–2 μg/μL. The liposome transfection reagent (Lipofectamine 3000) needs to be equilibrated at room temperature for 30 min before use. Prepare 2 sterile EP tubes and add 250 μL of serum-free medium to each respectively; add 2–5 μg of plasmid DNA to one tube and the liposome reagent to the other (with a mass ratio of plasmid to liposome ranging from 1:2 to 1:3). Gently mix each tube separately and let them stand at room temperature for 5 min. Slowly pour the plasmid dilution into the liposome dilution, gently pipette to mix, and let stand at room temperature for 20 min to form liposome-plasmid complexes. Directly add 500 μL of the complex mixture dropwise to the corresponding cell wells, gently shake the culture plate to ensure uniform distribution, and incubate at 37 °C, 5% CO_2_ for 4–6 h. After that, change the medium and continue culturing for 24–72 h to verify the transfection efficiency.

### 2.6 Animal model of intrauterine adhesion

All animal procedures were approved by the Animal Ethics Committee of Shanghai University of Traditional Chinese Medicine (Approval No. PZSHUTCM2404240002) and conducted in compliance with ARRIVE guidelines. Female Sprague-Dawley rats (8–10 weeks old, 200–220 g) were housed under controlled conditions (25 °C, 12 h light/dark cycle). In detail, rats were anesthetized with pentobarbital sodium (40 mg/kg, i. p.), and the uterine horns were exposed via midline laparotomy. A 95% ethanol-soaked cotton thread was inserted into the uterine lumen for 5 min to induce chemical injury, followed by gentle mechanical scraping with a sterile surgical blade. Sham-operated rats underwent laparotomy only. Postoperative analgesia (buprenorphine, 0.05 mg/kg) was administered for 48 h. Model success was confirmed by histological examination (HE and Masson staining) 7 days post-injury, characterized by endometrial thinning, glandular atrophy, and collagen deposition. The interval between modeling and drug treatment initiation was 24 h, allowing stabilization of the IUA phenotype.

### 2.7 Experimental design and grouping

*In vivo*: Sample size was determined based on previous studies and power analysis (α = 0.05, β = 0.2, effect size = 1.5) using G*Power 3.1, resulting in n = 10 per group. Rats were randomly assigned using a random number table to: (1) Sham group (sham operation + vehicle); (2) IUA group (model + vehicle); (3) IUA + Rubiadin (50 mg/kg, i. p., daily for 7 days); (4) IUA + Rubiadin + Dorsomorphin (25 mg/kg, i. p., daily). Drug administration began 24 h post-modeling. *In vitro*: hESCs were treated with Rubiadin (20, 40, 50 μM) or co-treated with Dorsomorphin (1 μM) for 24–48 h. Control groups received vehicle (DMSO ≤0.1%). Each experiment was independently repeated three times. Researchers were blinded to group allocation during data collection and analysis. During the assessment of animal pregnancy outcomes, a single-blind design was adopted: the laboratory personnel responsible for observing the pregnancy status were unaware of the grouping information of the rats and only recorded the results based on a unified pregnancy determination standard (vaginal thrombectomy observation + postoperative uterine embryo count).

### 2.8 Protein thermal shift assay (CETSA)

hESCs were treated with Rubiadin (50 μM) or vehicle for 4 h, lysed, and heated at 37, 40, 50, 60, and 70 °C for 3 min. Soluble proteins were analyzed by Western blot for AMPK and Piezo1. Thermal stability curves were plotted, and ΔTm values were calculated from three independent experiments.

### 2.9 TUNEL cell apoptosis detection experimental method

Slices were fixed with 4% paraformaldehyde at room temperature for 30 min, washed with PBS, then permeabilized with 0.1% Triton X-100 for 10–20 min to expose DNA cleavage sites. TUNEL reaction solution (enzyme + labeling solution, prepared per Biyun Tian C1088 kit instructions) was added, incubated at 37 °C in the dark for 60 min, then washed with PBS. Nuclei were stained with DAPI (as in IF step). After staining was completed, apoptotic cells (showing specific green fluorescence) were observed under a fluorescence microscope using a ×40 objective lens and quantitative analysis was conducted: Randomly select one section from each group of three animals. For each section, choose five non-overlapping fields (with a field range of 100 μm scale). The percentage of TUNel-positive cells in each field of view to the total cells is counted as a quantitative indicator of apoptosis level.

### 2.10 Acquisition of therapeutic targets for BSHX prescription in the treatment of IUA

Potential metabolites of BSHX were retrieved from the PubChem database, including their PubChem CID, SMILES notations, and 3D structural information, to serve as the basis for target prediction. Systematic prediction of protein targets for these metabolites was performed using three complementary platforms: the Comparative Toxicogenomics Database (CTD), SwissTargetPrediction, and PharmMapper. To enhance the reliability of predictions and mitigate potential false positives inherent to individual databases, we adopted a consensus strategy. Specifically, targets predicted by at least two independent methods were considered higher-confidence candidates and were selected to constitute the potential drug target set of BSHX for subsequent analysis. In parallel, disease-associated targets for IUA were collected from the GeneCards, DisGeNET, and CTD databases, yielding a total of 2,673 targets. The intersection between the high-confidence BSHX target set (“targets predicted by at least two methods”) and the IUA-related target set was then computed. The resulting overlapping targets were defined as the potential therapeutic targets through which BSHX may exert its effects against IUA, and were used for subsequent network construction and enrichment analysis.

### 2.11 Molecular dynamics simulations

To assess the binding stability of the key candidate metabolite Rubiadin with its potential targets, molecular dynamics (MD) simulations were performed on the Piezo1–Rubiadin and AMPK–Rubiadin complexes using GROMACS (version 2025.4). The initial structures of the protein–ligand complexes were obtained from molecular docking. The protein topology was generated with the pdb2gmx tool, and force field parameters for Rubiadin were assigned based on the GROMOS force field. Each complex was solvated in a cubic box with TIP3P explicit water molecules, and ions were added to neutralize the system and mimic physiological salt concentration. The systems underwent energy minimization followed by equilibration in the NVT (constant number, volume, and temperature) and NPT (constant number, pressure, and temperature) ensembles. Subsequently, production MD runs were carried out for 50 ns under periodic boundary conditions. The trajectories were saved every 10 ps for analysis. The stability of each complex was evaluated by calculating the root mean square deviation (RMSD) of the protein backbone and ligand, the root mean square fluctuation (RMSF) of protein residues, and the evolution of intermolecular hydrogen bonds over the simulation time. All analyses were conducted using built-in GROMACS tools and in-house scripts.

### 2.12 Functional assays

Cell viability (CCK-8), apoptosis (Annexin V-FITC/PI), migration (scratch assay), invasion (Transwell with Matrigel), mitochondrial membrane potential (JC-1), and LDH release were performed according to manufacturers’ protocols. For each assay, at least three biological replicates were included. ECM turnover markers (MMP-9, TIMP-1, hydroxyproline) were quantified by ELISA.

### 2.13 Western blot, qPCR, and immunofluorescence

Total proteins and RNA were extracted using RIPA lysis buffer and TRIzol, respectively. Western blot was performed with antibodies listed in Supplementary Table S1. Full-length uncropped blots are provided in Supplementary Material. qPCR was conducted with gene-specific primers (Supplementary Table S3) using SYBR Green on a QuantStudio 5 system. Immunofluorescence was performed on paraffin-embedded sections or fixed cells. Images were quantified using Image-Pro Plus 6.0. This study strictly implemented randomization and blinding operations throughout the experiment to control bias: Animal grouping was completed using the random number table method for fully random allocation. Cell culture Wells were randomly designed and allocated based on cell batches as block factors. Before quantitative analysis of histological sections and WB bands, de-identification coding was carried out and grouping information was sealed. Result evaluation was conducted by two independent individuals who were not familiar with sample grouping in a double-blind manner, and the final mean was taken for statistics.

### 2.14 Histopathology and pregnancy assessment

For clinical samples, HE staining was observed under a ×10 objective lens to examine the overall tissue structure (corresponding to a 100 μm scale bar), and typical regions were selected for detailed observation under a ×40 objective lens (corresponding to a 20 μm scale bar). For animal models, Masson staining was observed under a ×10 objective lens (corresponding to a 100 μm scale bar), and the collagen area fraction (the proportion of collagen-positive areas to the total area of the visual field) was quantitatively analyzed using ImageJ software. Immunofluorescence staining was observed under a ×10 objective lens (corresponding to a 100 μm scale bar), and the percentage of caspase 3-positive cells in each visual field relative to the total number of cells was counted. In the quantitative analysis, for animal models, 1 section from 3 animals in each group was selected, and 5 non-overlapping visual fields were randomly chosen from each section. For clinical samples, 1 section from 1 sample was selected, and 4 typical visual fields were chosen from each section. Finally, the mean value of the visual fields in each group was used as the statistical result.

Uterine tissues were processed for HE and Masson staining. Collagen area fraction was quantified using ImageJ.

For mating experiments, treated female rats were co-housed with fertile males (1:3). Pregnancy rate, implantation sites, and litter size were recorded. Statistical analysis of pregnancy rates was performed using Fisher’s exact test. Researchers were blinded to group allocation during outcome assessment.

### 2.15 Statistical analysis

Data are presented as mean ± standard deviation (SD) or mean ± standard error of the mean (SEM), as specified in individual figure legends. The normality of data distribution was assessed using the Shapiro-Wilk test. For comparisons between two independent groups: unpaired two-tailed Student’s t-test was used for parametric data, while the Mann–Whitney U test was applied for non-parametric data. For comparisons across multiple groups: one-way analysis of variance (ANOVA) was performed first, followed by Tukey’s *post hoc* test for pairwise comparisons. Pregnancy rates were analyzed using Fisher’s exact test, given the categorical nature of the outcome.

Pregnancy rate (number of pregnant female mice/total number of mated female mice, expressed as a percentage); in the statistical analysis, the pregnancy rate was compared between groups using Fisher’s exact test.

## 3 Results

### 3.1 Intrauterine adhesion tissues inhibit cell apoptosis through the AMPK-p53-p21 pathway

**FIGURE 2:**
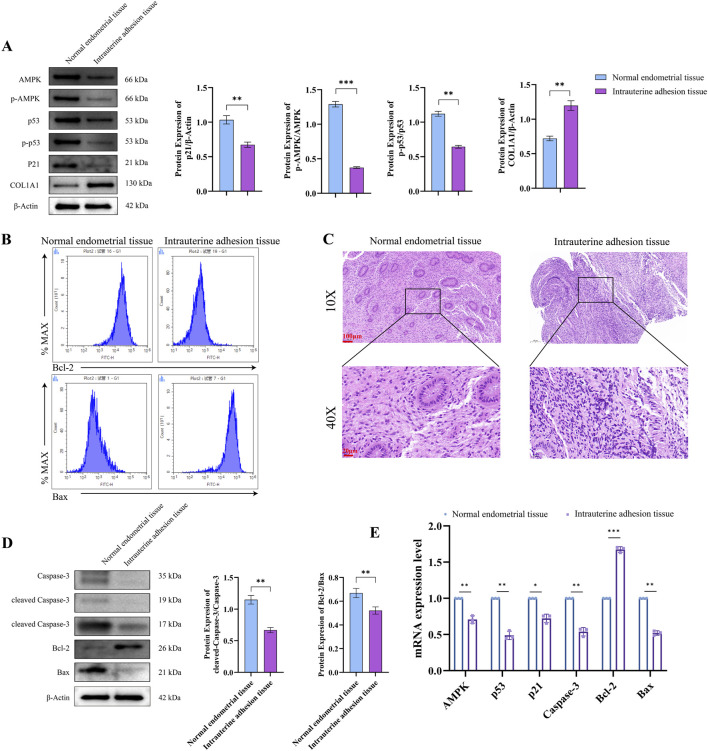
Intrauterine Adhesion Tissues Inhibit Cell Apoptosis Through the AMPK-p53-p21 Pathway **(A)** Representative Western blot bands and quantitative analysis of AMPK, p-AMPK, p53, p-p53, p21, and COL1A1 protein levels in normal endometrial tissue and intrauterine adhesion (IUA) tissue. Sample size (n = 3) per group; statistical analysis: unpaired two-tailed t-test; data are presented as mean ± SD. **(B)** Flow cytometry analysis of apoptosis-related proteins Bcl-2 and Bax in normal endometrial tissue and IUA tissue. Sample size (n = 3) per group; statistical analysis: Mann–Whitney U test (non-parametric data); data are presented as mean ± SEM. **(C)** Hematoxylin-eosin (HE) staining of normal endometrial tissue and IUA tissue. Scale bar = 100 μm; representative images from (n = 3) biological replicates. **(D)** Western blot verification of AMPK, p53, p21, Caspase-3, Bcl-2, and Bax protein expression levels in normal endometrial tissue and IUA tissue. Sample size (n = 3) per group; statistical analysis: unpaired two-tailed t-test; data are presented as mean ± SD. **(E)** QPCR analysis of AMPK, p53, p21, Caspase-3, Bcl-2, and Bax mRNA expression levels in normal endometrial tissue and IUA tissue. Sample size (n = 3) per group; statistical analysis: unpaired two-tailed t-test; data are presented as mean ± SD.

To elucidate the pathogenesis of IUA and identify potential therapeutic targets of the *BSHX*, we conducted a comprehensive comparison of molecular expression profiles using clinical tissue samples from patients with IUA and normal endometrial controls. Western blot analysis revealed significant downregulation of phosphorylated AMPK (p-AMPK), phosphorylated p53 (p-p53), and p21 protein expression in IUA tissues compared to normal controls (*P* < 0.05), concomitant with marked upregulation of COL1A1 extracellular matrix protein (*P* < 0.05; Figure 2A). IUA tissues exhibited pronounced downregulation of AMPK, p53, p21, Caspase-3 and Bax mRNA levels (*P* < 0.05), with p53 showing the most dramatic reduction. Conversely, anti-apoptotic Bcl-2 expression was significantly elevated (*P* < 0.05). Further observation revealed an increase in Bcl-2 expression and a decrease in Bax expression (Figure 2B). In addition, Histological examination via HE staining demonstrated characteristic IUA features, including endometrial thinning, glandular atrophy, and severe fibrotic adhesions. High-power magnification (×40) revealed disorganized cellular architecture and prominent fibrous tissue hyperplasia (Figure 2C). Western blot confirmation showed concordant protein-level changes: upregulated Bcl-2 and downregulated Caspase-3, cleaved caspase-3, and Bax (Figure 2D). Finally, The qPCR analysis further revealed significant alterations in apoptosis-related gene expression (Figure 2E). These findings demonstrate substantial inhibition of the AMPK-p53-p21 signaling pathway in IUA, coupled with excessive extracellular matrix deposition, suggesting that dysregulation of this pathway and ECM homeostasis may contribute to IUA pathogenesis.

Collectively, these findings indicate that, apoptosis is inhibited via downregulation of the AMPK-p53-p21 pathway in IUA tissues, representing a fundamental mechanism in IUA pathogenesis. The demonstrated interactions between Rubiadin and key pathway targets suggest potential therapeutic avenues for IUA treatment.

### 3.2 Integrated chemical and multi-target network analysis reveals the mechanism of BSHX in treating intrauterine adhesions via modulating the AMPK signaling pathway

**FIGURE 3:**
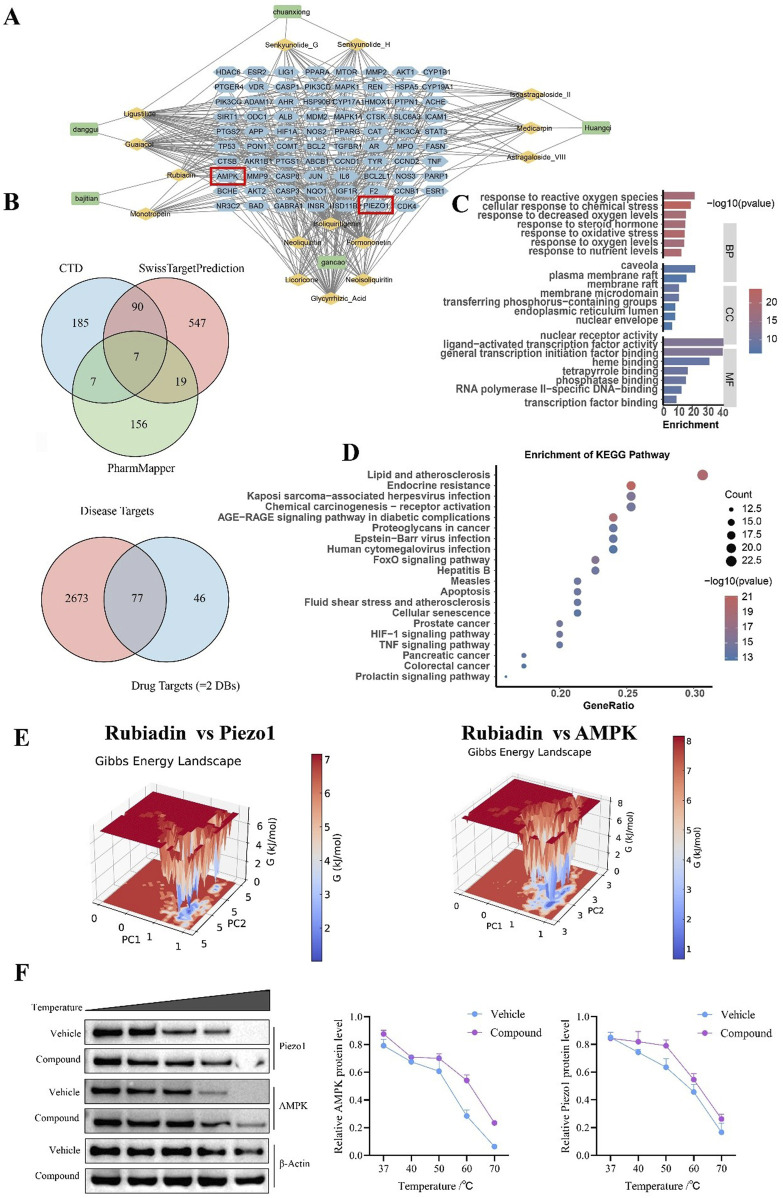
Integrated Chemical and Multi-Target Network Analyses Reveal BSHX’s Therapeutic Mechanisms in Intrauterine Adhesions. **(A)** The predicted target protein interaction (PPI) network of the core metabolite Rubiadin in BSHX demonstrates the potential interaction relationships between Rubiadin’s target proteins, with AMPK and Piezo1 identified as core hub genes (highlighted in red boxes) **(B)** The Venn diagram above illustrates the intersection of targets predicted by three databases: CTD, SwissTargetPrediction, and PharmMapper. The Venn diagram below shows the intersection of disease targets and drug targets, resulting in a total of 77 core targets. **(C)** GO functional enrichment analysis. From the three perspectives of biological process (BP), cellular metabolite (CC), and molecular function (MF), it displays the significantly enriched functional entries of core targets, with the color intensity representing the -log10 (pvalue) value. **(D)** KEGG pathway enrichment analysis bubble chart. It displays the signaling pathways significantly enriched by core targets. The x-axis represents the gene enrichment ratio (GeneRatio), the size of the bubble indicates the number of genes, and the color corresponds to the -log10 (pvalue) value. **(E)** Gibbs energy landscape diagram of Rubiadin binding with Piezo1 and AMPK. The three-dimensional diagram illustrates the conformational energy distribution of the complex during molecular dynamics simulations, with red areas representing states of lower energy and more stable conformations. **(F)** Western blot analysis of Piezo1 and AMPK protein levels under different temperature conditions (Vehicle vs. Metabolite treatment).

To characterize the chemical profile of BSHX, we first established a candidate metabolite pool of 374 metabolites from public databases (TCMIP 2.0, TCMSP) and literature. UHPLC-Q-Orbitrap HRMS analysis confirmed the presence of 96 major metabolites in BSHX extract (Supplementary Figure S1A). By comparing the predicted candidates with experimentally identified metabolites, Venn analysis revealed 15 overlapping metabolites, which were designated as core metabolites and selected for subsequent target prediction. Potential protein targets for these 15 metabolites were predicted using three complementary databases (CTD, SwissTargetPrediction, PharmMapper). Venn analysis showed 7 targets common to all three methods and 123 targets predicted by at least two methods. Recognizing the potential for false positives inherent to such predictive databases, we adopted a conservative approach by focusing on the 123 targets identified by ≥2 methods as a higher-confidence candidate target set for BSHX. Separately, 2,673 IUA-related targets were aggregated from disease databases (GeneCards, DisGeNET, CTD). The intersection of these two sets yielded 77 overlapping targets, which were hypothesized as potential therapeutic targets linking BSHX metabolites to IUA pathology (Figure 3B).

To further investigate the biological relevance of these 77 intersecting targets, we performed KEGG pathway enrichment analysis. The results highlighted pathways critically implicated in fibrotic diseases and tissue repair, including endocrine resistance, the AGE-RAGE signaling pathway, the PI3K-Akt pathway, and cellular senescence—all of which are known contributors to IUA pathogenesis (Figure 3C). GO analysis further suggested the involvement of biological processes such as stress response and phosphorylation-related signaling (Figure 3D).

Among the 15 core metabolites, Rubiadin was selected for in-depth protein-protein interaction (PPI) network analysis due to its high relative abundance in BSHX extract and its potential pharmacological relevance. PPI network analysis of Rubiadin’s predicted targets revealed a complex interaction network, with AMPK and Piezo1 identified as core hub genes (Figure 3A). Notably, these hub genes are closely linked to the enriched pathways regulating inflammation, metabolism, and cellular stress responses, suggesting their potential centrality in BSHX’s therapeutic effects against IUA. To preliminarily assess the feasibility of key metabolite-target interactions suggested by the network, we conducted molecular dynamics simulations on Rubiadin, one of the core metabolites of BSHX, with two representative targets (AMPK and Piezo1) implicated in the enriched pathways. The simulations suggested that Rubiadin could form stable binding poses with both proteins, as indicated by favorable binding energies, minimal conformational fluctuation during dynamics, and enhanced thermal stability in the case of Piezo1 (Figure 3E; Supplementary Figures S1B–G). To further validate Rubiadin’s binding to Piezo1 and AMPK, we performed replicate CETSA (Figure 3F), assessing their soluble abundance across 37 °C–70 °C in Rubiadin-treated groups. For Piezo1 (286 kDa), soluble protein persisted at 60 °C–70 °C; for AMPK (63 kDa), higher soluble abundance was retained at 50 °C–70 °C (two technical replicates per target, consistent trends) (Supplementary Figure S2). Collectively, this integrated *in silico* and preliminary experimental approach established a rational basis for prioritizing Rubiadin and the AMPK signaling pathway for subsequent experimental validation. The computational predictions, while requiring further functional confirmation, provided a robust foundation for exploring the mechanistic role of BSHX in ameliorating IUA.

### 3.3 Rubiadin promotes apoptosis of endometrial stromal cells in a dose-dependent manner

**FIGURE 4:**
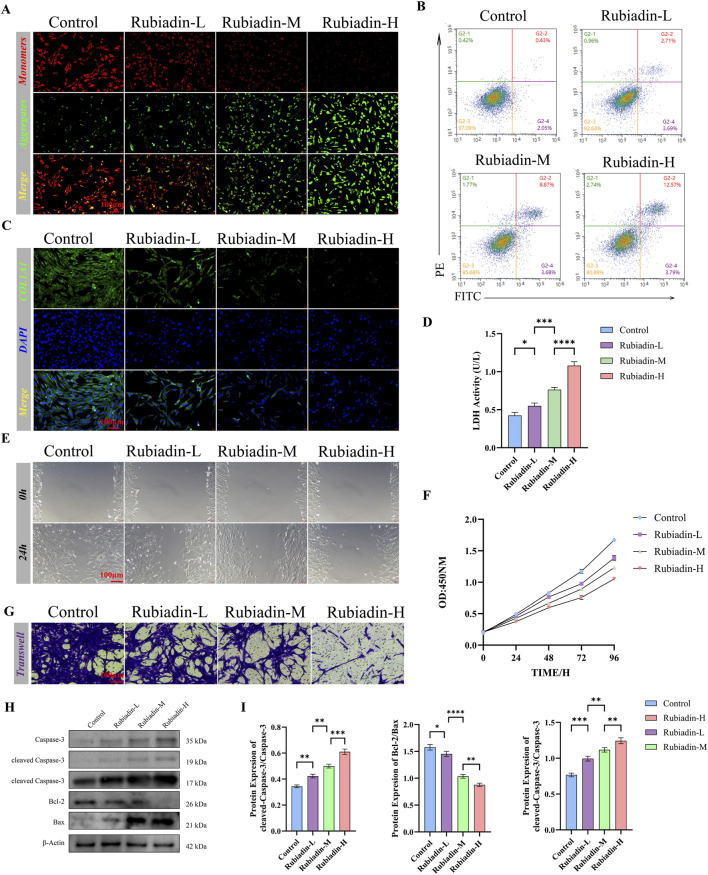
Rubiadin Promotes Apoptosis of Endometrial Stromal Cells in a Dose-Dependent Manner **(A)** JC-1 staining showed changes in mitochondrial membrane potential of endometrial stromal cells treated with different concentrations of Rubiadin (Rubiadin-L, - M, - H) and control. Quantitative analysis of red/green fluorescence ratio was displayed (scale: 100 μm). Sample size \(n = 3\) cell experimental replicates; statistical analysis: one-way ANOVA + Tukey’s *post hoc* test; data presented as mean ± SEM. **(B)** Flow cytometry analysis of endometrial stromal cell apoptosis after treatment with different doses of Rubiadin (Annexin V-FITC/PI staining). Sample size (n = 3) cell experimental replicates; statistical analysis: one-way ANOVA + Tukey’s *post hoc* test; data presented as mean ± SEM. **(C)** Immunofluorescence staining of COL1A1 (green) and DAPI (blue) in endometrial stromal cells (scale bar: 50 μ m). Sample size (n = 3) cell experimental replicates. **(D)** The LDH release assay showed the cytotoxic effects of different Rubiadins on endometrial stromal cells. Injury. Statistical analysis: one-way ANOVA + Tukey’s *post hoc* test; data presented as mean ± SEM. **(E)** Cell scratch assay: Migration ability of endometrial stromal cells treated with Rubiadin for 0 and 24 h (scale: 100 μm). Statistical analysis: one-way ANOVA + Tukey’s *post hoc* test; data presented as mean ± SEM. **(F)** The CCK-8 assay showed a dose - and time-dependent effect of Rubiadin on the survival rate of endometrial stromal cells. Statistical analysis: two-way ANOVA + Tukey’s *post hoc* test; data presented as mean ± SEM. **(G)** Transwell invasion assay showed the invasive ability of endometrial stromal cells treated with different doses of Rubiadin. (Scale: 100 μm). Statistical analysis: one-way ANOVA + Tukey’s *post hoc* test; data presented as mean ± SEM. **(H)** Western blot analysis of apoptosis related proteins:Caspase-3, cleaved Caspase-3 (19 kDa and 17 kDa), Bcl-2, Bax in endometrial stromal cells treated with Rubiadin. β - actin is used as an internal reference. Sample size (n = 3) biological replicates; statistical analysis: one-way ANOVA + Tukey’s *post hoc* test; data presented as mean ± SEM. **(I)** Quantification of protein expression levels of cleaved Caspase 3, Bax, Bcl-2, and cleaved Caspase-3/Bcl-2 ratio in different groups. Sample size (n = 3) biological replicates; statistical analysis: one-way ANOVA + Tukey’s *post hoc* test; data presented as mean ± SEM. Compared with the control group, the values are expressed as mean ± SEM (n = 3 in cells), *p < 0.05, **p < 0.01, **p < 0.001.

Next, *in vitro* experiments will focus on the metabolite Rubiadin in the kidney tonifying and blood activating formula. Different concentration treatment groups (Rubiadin-L, Rubiadin-M, Rubiadin-H) and control groups will be established to elucidate the role of Rubiadin in the intervention of endometrial adhesion from the aspects of cell survival rate, apoptosis process, migration and invasion ability, and related molecular expression. Quantitative analysis of JC-1 staining demonstrated that Rubiadin treatment induced dose-dependent mitochondrial depolarization, characterized by a progressive shift from red to green fluorescence and a corresponding increase in early apoptotic cell populations (*P* < 0.05, Figure 4A).

Notably, Rubiadin induced significant apoptosis in a concentration-dependent manner (Figure 4B). Immunofluorescence analysis showed that Rubiadin significantly inhibited COL1A1 expression in a concentration-dependent manner (*P* < 0.05; Figure 4C), indicating its potential to alleviate extracellular matrix deposition. LDH release assays confirmed its cytotoxic effects, with progressively greater cellular damage observed at higher concentrations (*P* < 0.05; Figure 4D). Functional assays further revealed multifaceted dose-dependent inhibitory effects of Rubiadin: it suppressed cell migration (Figure 4E), inhibited proliferation in both dose- and time-dependent manners (Figure 4F), and reduced invasive capacity with increasing concentrations (Figure 4G). Western blot analysis further revealed a concentration-dependent upregulation of pro-apoptotic proteins, including Caspase-3, cleaved Caspase-3, and Bax, accompanied by downregulation of the anti-apoptotic protein Bcl-2 (*P* < 0.05) (Figures 4H,I).

These comprehensive findings collectively demonstrate that Rubiadin exerts inhibitory effects on fibrotic and proliferative processes while promoting apoptotic pathways in endometrial stromal cells. In summary, Rubiadin regulates cell apoptosis and functional behaviors by dose-dependently activating the AMPK-p53-p21 pathway. To further verify the anti-fibrotic activity of Rubiadin, we evaluated extracellular matrix (ECM) renewal and collagen deposition markers. Western blot and quantitative analysis revealed that Rubiadin regulated ECM dynamics in a dose-dependent manner: compared with the control group, Rubiadin treatment significantly upregulated the expression of matrix metalloproteinase-9 (MMP-9), while its inhibitor TIMP-1 was downregulated in a dose-dependent manner (Supplementary Figure S3). These results indicate that Rubiadin promotes ECM degradation and reduces collagen deposition, which supplements its inhibitory effect on COL1A1 expression and further supports its anti-fibrotic effect in endometrial stromal cells.

### 3.4 Rubiadin promotes apoptosis of endometrial stromal cells by activating the AMPK-p53-p21 pathway

**FIGURE 5:**
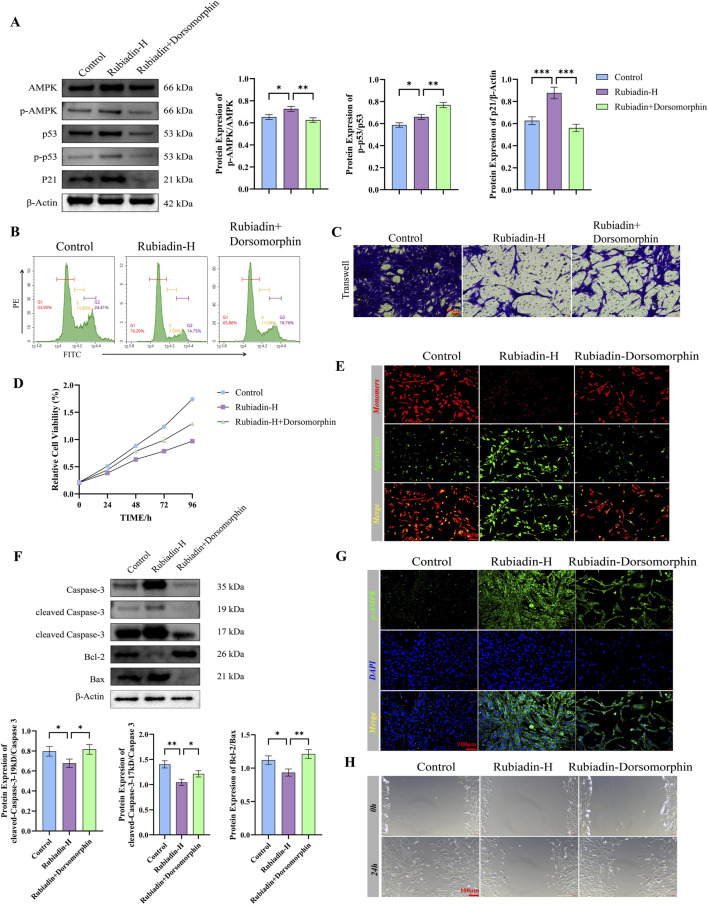
Rubiadin Promotes Apoptosis of Endometrial Stromal Cells by Activating the AMPK-p53-p21 Pathway **(A)** Western blot analysis and quantification of AMPK, phosphorylated AMPK (p - AMPK), p53, phosphorylated p53 (p - p53), and p21 protein levels in Control, Rubiadin - H, and Rubiadin + Dorsomorphin groups. Sample size (n = 3) biological replicates; statistical analysis: one-way ANOVA + Tukey’s *post hoc* test; data presented as mean ± SEM. **(B)** Flow cytometry analysis of cell cycle distribution of endometrial stromal cells. Statistical analysis: one-way ANOVA + Tukey’s *post hoc* test; data presented as mean ± SEM. **(C)** Transwell invasion assay showed the invasive ability of Rubiadin-H and Dorsomorphin in treating or not treating endometrial stromal cells. (Scale: 100 μm). Sample size (n = 3) cell experimental replicates; statistical analysis: one-way ANOVA + Tukey’s *post hoc* test; data presented as mean ± SEM. **(D)** Cell survival curves of endometrial stromal cells treated/untreated with Rubiadin-H and Dorsoporphin at 0, 24, 48, 72, and 96 h. Sample size (n = 3) cell experimental replicates; statistical analysis: two-way ANOVA + Tukey’s *post hoc* test; data presented as mean ± SEM. **(E)** JC-1 staining showed changes in mitochondrial membrane potential (scale bar: 100 μm). Sample size (n = 3) cell experimental replicates; statistical analysis: one-way ANOVA + Tukey’s *post hoc* test; data presented as mean ± SEM. **(F)** Western blot and quantitative analysis of Caspase-3, cleaved Caspase-3 (19 kDa and 17 kDa), Bcl-2, and Bax in endometrial stromal cells. Sample size (n = 3) biological replicates; statistical analysis: one-way ANOVA + Tukey’s *post hoc* test; data presented as mean ± SEM. **(G)** Immunofluorescence staining of p-AMPK (green) and DAPI (blue, nuclear staining) in endometrial stromal cells (scale bar: 100 μm). Sample size (n = 3) cell experimental replicate. **(H)** Cell scratch assay showed the migration ability of endometrial stromal cells (scale: 100 μm). Statistical analysis: one-way ANOVA + Tukey’s *post hoc* test; data presented as mean ± SEM. Compared with the control group, the values are expressed as mean ± SEM (n = 3 in cells), *p < 0.05, **p < 0.01, **p < 0.001.

To elucidate the molecular mechanism of Rubiadin’s therapeutic effect, we employed the specific AMPK inhibitor Dorsomorphin for pharmacological inhibition. Our results indicate that the combination therapy of Dorsomorphin effectively attenuated the activation of the Rubiadin mediated AMPK-p53-p21 pathway, as evidenced by significant reductions in the expression levels of phosphorylated AMPK (p-AMPK), phosphorylated p53 (p-p53), and p21 protein. Immunofluorescence results also confirmed that AMPK expression was inhibited by Dorsomorphin (Figures 5A,G). Cell cycle analysis showed that high-dose Rubiadin (Rubiadin-H) induced significant G1 phase arrest (*P* < 0.05), and the combination therapy of Dorsomorphin significantly reversed this effect (*P* < 0.05; Figure 5B). In addition, Rubiadin-H treatment significantly impaired cell invasiveness, migration ability, and survival rate, all of which were partially rescued by Dorsomorphin combination therapy (Figures 5C,D,H), clearly indicating the dependent regulation of AMPK on these key cellular processes. At the subcellular level, Rubiadin-H induces mitochondrial dysfunction characterized by depolarization of membrane potential and increased apoptotic signals, which was partially improved by treatment with Dorsomorphin (Figure 5E). Molecular analysis confirmed that Dorsomorphin counteracts Rubiadin’s pro apoptotic effect by coordinating the downregulation of pro apoptotic effectors and upregulation of anti apoptotic Bcl-2 (Figure 5F).

To clarify the time-dependent activation of the AMPK/p53/p21 pathway by Rubiadin, we further conducted a time-history analysis (0, 6, 12, 24 h) of pae-related proteins in stromal cells treated with Rubiadin-H. Western blot and quantitative results showed (Supplementary Figure S4). Compared with the control group, the continuous activation of the Rubiadin-H treatment-induced pathway: the level of p-AMPK rose for the first time at 6 h, then p-p53 gradually upregulated (reaching the peak at 12 h), and then the expression of p21 increased (lasting for 24 h). This time-dependent activation pattern directly supports that p53 phosphorylation is a downstream event of AMPK activation in these stromal cells, further verifying the causal relationship of the AMPK-p53-P21 axis.

To further verify AMPK-dependency (as suggested by the dependency of Rubiadin’s actions), we performed AMPK gene knockdown experiments using shRNA. We set four groups: Control, Rubiadin-H, shNC + Rubiadin-H, and shAMPK + Rubiadin-H. Western blot analysis revealed that: compared with the shNC + Rubiadin-H group, p-AMPK, p-p53, and p21 protein levels were significantly decreased in the shAMPK + Rubiadin-H group, while total AMPK and p53 levels showed no obvious changes, and the level of apoptosis decreases (Supplementary Figure S5). These results indicate that knockdown of AMPK blocks Rubiadin-induced activation of the p53-p21 pathway.

The demonstrated AMPK-dependency of Rubiadin’s actions provides strong mechanistic validation for its therapeutic application in intrauterine adhesion treatment, particularly through targeted activation of the AMPK-p53-p21 pathway in endometrial stromal cells. These findings have important implications for developing more precise therapeutic strategies for fibrotic uterine conditions.

### 3.5 Rubiadin promotes apoptosis of endometrial stromal cells and improves intrauterine adhesions *in Vivo* by activating the AMPK-p53-p21 pathway

**FIGURE 6:**
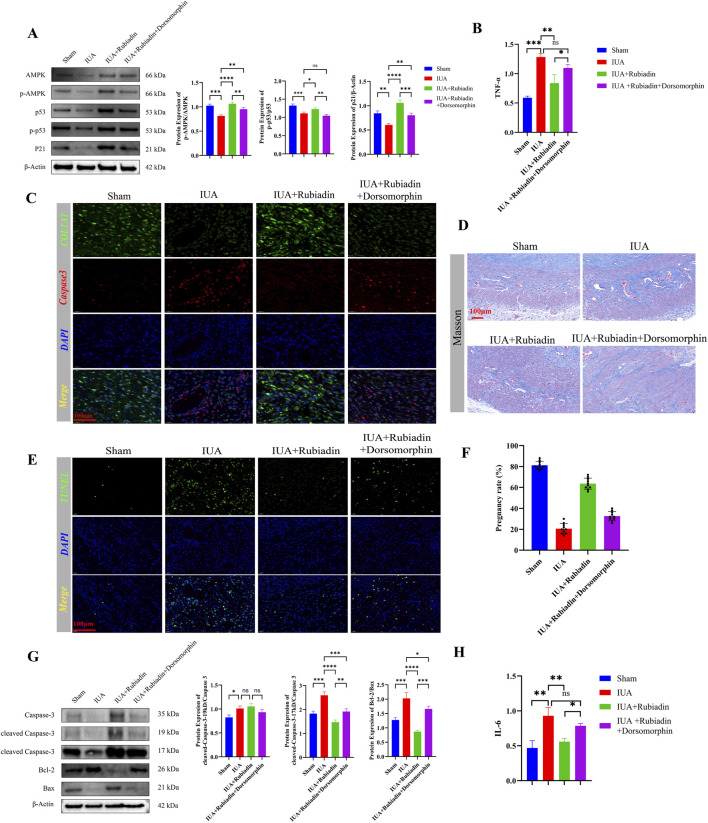
Rubiadin Promotes Apoptosis of Endometrial Stromal Cells and Improves Intrauterine Adhesions *in Vivo* by Activating the AMPK-p53-p21 Pathway **(A)** Protein blotting of p-AMPK, p-p53, and p21 in uterine tissues of Sham group, IUA group, IUA + Rubiadin group, and IUA + Rubiadin + Dorsomorphin group. β - actin is used as an internal reference. Quantitative analysis is presented as a scatter plot. Sample size (n = 3) rats per group; statistical analysis: one-way ANOVA + Tukey’s *post hoc* test; data presented as mean ± SEM. **(B)** Elisa detects serum TNF - α levels in different groups. Sample size (n = 3) rats per group; statistical analysis: one-way ANOVA + Tukey’s *post hoc* test; data presented as mean ± SEM. **(C)** Immunofluorescence co staining of Caspase-3 (red) and COL1A1 (green) in endometrial tissues from different groups, with DAPI (blue) used for nuclear staining. (Scale: 100 μm). Sample size\(n = 1) rats per group. **(D)** Masson staining in different groups is used to display collagen fiber deposition (blue). (Scale: 100 μm). Sample size\(n = 1) rats per group. **(E)** TUNEL detection (green) and DAPI (blue) nuclear staining of apoptotic cells in different groups. (Scale: 100 μm). Sample size\(n = 1) rats per group. **(F)** The pregnancy rates of each group are presented in bar chart format. Sample size (n = 8) mated rats per group; statistical analysis: Fisher’s exact test. **(G)** Western blot analysis of Caspase-3, cleaved Caspase-3, Bcl-2, Bax in different groups of uterine tissues. (β - actin as a reference). Sample size (n = 3) rats per group; statistical analysis: one-way ANOVA + Tukey’s *post hoc* test; data presented as mean ± SEM. **(H)** Elisa detects serum IL-6 levels in different groups. Sample size (n = 3) rats per group; statistical analysis: one-way ANOVA + Tukey’s *post hoc* test; data presented as mean ± SEM. Compared with the sham group, the values are expressed as mean ± SEM (n = 3 in rats), *p < 0.05, **p < 0.01, **p < 0.001.

To evaluate the therapeutic efficacy of Rubiadin *in vivo*, a rat model of intrauterine adhesion (IUA) was established and subjected to comprehensive analysis. Western blot results indicated that Rubiadin treatment effectively activated the AMPK-p53-p21 pathway in IUA rats, as shown by increased expression of p-AMPK, p-p53, and p21. However, the application of Dorsomorphin (an AMPK inhibitor) significantly attenuated these activating effects (Figure 6A). ELISA assays showed that Rubiadin markedly reduced the elevated levels of inflammatory factors TNF-α and IL-6 in IUA model rats, while co-treatment with Dorsomorphin notably diminished this therapeutic effect (*P* < 0.05; Figures 6B,H). Immunofluorescence analysis revealed that Rubiadin promoted Caspase-3 expression and suppressed COL1A1, effects that were also weakened upon Dorsomorphin intervention (*P* < 0.05; Figure 6C). Masson staining demonstrated that Rubiadin alleviated endometrial fibrosis and reduced collagen deposition, whereas the addition of Dorsomorphin partially reversed these improvements (*P* < 0.05; Figure 6D). Functional assessment showed that Rubiadin improved the reproductive outcomes of IUA rats. The pregnancy rate of the Sham group was approximately 80%, with 8 fertile female rats and 2 infertile female rats. The pregnancy rate of the IUA group was approximately 20%, with 2 fertile female mice and 8 infertile female mice. The pregnancy rate of the IUA + Rubiadin group was approximately 60%, with 6 fertile female mice and 4 infertile female mice. Compared with the IUA group, the pregnancy rate was significantly increased (*P < 0.05*; Figure 6F). The gestation rate of IUA + Rubiadin + Dorsomorphin was approximately 30%, with 3 fertile female mice and 7 infertile female mice (Figures 6E,G). These findings collectively suggest that the therapeutic benefits of Rubiadin in IUA are largely mediated through AMPK-dependent mechanisms.

## 4 Discussion

In recent years, the incidence of IUA has been increasing, seriously endangering the reproductive health of women of childbearing age (Asherman JG,0.1948). Treatments for IUA include endometrial adhesion incision, intrauterine device placement, and estrogen therapy (Healy M. W. et al., 2016). However, functional reconstruction of moderate to severe endometrial damage remains a significant challenge (Xiao S. et al., 2014). As a refractory complication following endometrial injury, IUA is thus plagued by persistent clinical dilemmas, particularly its high recurrence rate and poor fertility outcomes in affected patients (Zeng P. et al., 2023; Zhao J. et al., 2023). Certain progress has been made in the research on TCM treatment of intrauterine adhesions. Many physicians believe that the basic pathogenesis of intrauterine adhesions is deficiency in origin and excess in superficiality, with kidney deficiency as the root cause and blood stasis as the superficial symptom. Therefore, tonifying the kidney and activating blood circulation has become the main treatment method for this disease. This study systematically clarified the mechanism by which Rubiadin, an metabolite of the Bushen Huoxue formula, promotes endometrial stromal cell apoptosis and inhibits collagen deposition through the AMPK-p53-p21 axis, providing experimental basis for TCM intervention in intrauterine adhesions.

This study reveals the critical role of the AMPK-p53-p21 pathway in the pathogenesis of intrauterine adhesions. Clinical sample analysis showed that compared with normal endometrial tissues, the expressions of AMPK, p53, p21, and apoptosis-related proteins in intrauterine adhesion tissues were significantly decreased, while the expressions of anti-apoptotic protein Bcl-2 and fibrosis marker COL1A1 were increased. This finding provides a new perspective for understanding the molecular mechanism of intrauterine adhesions. As a core regulator of cellular energy metabolism, the decreased activity of AMPK leads to the inhibition of the p53-p21 signaling pathway, thereby reducing endometrial stromal cell apoptosis and promoting the fibrosis process, which is consistent with the “apoptosis escape-fibrosis” model of myocardial ischemia/reperfusion (I/R) injury (Xin L. et al., 2021). It is worth noting that the mRNA downregulation of Caspase-3 was the most significant, suggesting that the silencing of the AMPK axis may inhibit the mitochondrial apoptotic pathway, keeping endometrial stromal cells in a long-term “anti-apoptotic” state, resulting in poor repair of the basal layer. At the same time, the increased levels of inflammatory factors TNF-α and IL-6 suggest that the inflammatory response may exacerbate this pathological process, forming a vicious cycle of “apoptosis inhibition-inflammation aggravation-fibrosis”.

This study clarifies the multi-target therapeutic mechanism of Rubiadin. Through network pharmacology and molecular docking technology, we found that metabolites such as Astragaloside VIII and Isoastragaloside II in the Bushen Huoxue formula can stably bind to targets such as AMPK and Piezo1. *In vitro* experiments confirmed that Rubiadin could dose-dependently activate the AMPK-p53-p21 pathway, promote the apoptosis of endometrial stromal cells, and inhibit cell migration, invasion, and COL1A1 expression, suggesting that Rubiadin has dual effects of promoting apoptosis and improving matrix metabolism, which cross-validates with the mechanism that AMPK activation can inhibit collagen deposition. Particularly importantly, the intervention experiment with the AMPK inhibitor Dorsomorphin clarified the pathway dependence. These findings not only clarify the molecular basis of the “activating blood circulation and resolving blood stasis” effect of the Bushen Huoxue formula but also provide an example for modern TCM research.

Finally, *in vivo* experiments completed the transformation from mechanism to efficacy, verifying the therapeutic potential of Rubiadin. In the intrauterine adhesion rat model, Rubiadin significantly improved the endometrial pathological state, reduced the levels of inflammatory factors, and increased the pregnancy rate. Dorsomorphin not only blocked the pro-apoptotic effect of Rubiadin but also partially restored COL1A1 expression and the levels of inflammatory factors TNF-α and IL-6, suggesting that the AMPK axis is the core target for Rubiadin to exert “anti-fibrosis-promoting fertility” benefits. In addition, the regulation of the inflammatory microenvironment by Rubiadin may be related to the inhibition of NF-κB signaling by AMPK, providing a supplementary mechanism for explaining its “anti-inflammatory-anti-fibrosis” synergistic effect. In this study, Dorsomorphin was used to inhibit the AMPK pathway to investigate its function; however, Dorsomorphin has a potential risk of target non-specificity (such as inhibitory effects on other kinases) (Chen et al., 2023). To further clarify the specific role of AMPK in Rubiadin’s regulation of endometrial stromal cell functions, subsequent studies will introduce the AMPK gene knockdown (shRNA) strategy to downregulate the expression of endogenous AMPK. The treatment experiment of Rubiadin + Dorsomorphin will be repeated in the knockdown cell model: if both the effect of Rubiadin and the inhibitory effect of Dorsomorphin are significantly reduced after AMPK knockdown, it can verify that the AMPK pathway is the specific target through which Rubiadin exerts its effects.

In addition, this study has certain limitations. The number of clinical samples is relatively limited, and future studies need to expand the sample size and conduct multi-center verification. The *in vivo* metabolic process of Rubiadin has not been clarified, and there is a lack of pharmacokinetic data to support its administration method, metabolic half-life, and tissue distribution characteristics, making it difficult to directly guide the design of clinical medication regimens. In addition, the animal model uses chemical injury to construct the IUA model, which has pathological differences from iatrogenic injury (such as uterine cavity operation) in human intrauterine adhesions, and the clinical transformation value of efficacy evaluation needs to be carefully evaluated.

## 5 Conclusion

Rubiadin promotes the apoptosis of endometrial stromal cells by activating the AMPK/p53/p21 pathway, improves the pathology of intrauterine adhesions and pregnancy outcomes, and provides potential targets and drugs for the treatment of intrauterine adhesions with traditional Chinese medicine.

## Data Availability

The data presented in the study are deposited in the iProX repository, accession ID IPX0016647000, available at: https://www.iprox.cn/page/project.html?id=IPX0016647000.

## Glossary

AMPK: AMP-activated protein kinase
BSHX: Bushen Huoxue formula
BCA: Bicinchoninic Acid
Bax: BCL2-Associated X Protein
BP: Biological process
Caspase-3: Cysteine-Aspartic Acid Protease 3
CC: Cellular Metabolite
CCK-8: Cell Counting Kit-8
COL1A1: Collagen Type I Alpha 1 Chain
CTRP6: C1q/Tumor Necrosis Factor-Related Protein-6
DAPI: 4′,6-Diamidino-2-Phenylindole
ECM: Extracellular matrix
ELISA: Enzyme-linked immunosorbent assay
FBS: Fetal bovine serum
GO: Gene Ontology
HE: Hematoxylin and eosin
HRP: Horseradish peroxidase
HRMS: High-resolution mass spectrometry
IF: Immunofluorescence
IL-6: Interleukin-6
IUA: Intrauterine adhesion
KEGG: Kyoto Encyclopedia of Genes and Genomes
LDH: Lactate dehydrogenase
MF: Molecular Function
OD: Optical density
p53: Tumor Protein 53
p21: Cyclin-Dependent Kinase Inhibitor 1A
PBS: Phosphate-buffered saline
PI: Propidium iodide
PVDF: Polyvinylidene fluoride
qPCR: Quantitative real-time polymerase chain reaction
SD: Sprague-Dawley (rats); Standard deviation
SDS-PAGE: Sodium dodecyl sulfate–polyacrylamide gel electrophoresis
TCRA: Transcervical Resection of Adhesion
TCM: Traditional Chinese Medicine
TCMIP: Integrated Pharmacology Platform of Traditional Chinese Medicine
TCMSP: Traditional Chinese Medicine Systems Pharmacology Database and Analysis Platform
TNF-α: Tumor Necrosis Factor-alpha
TUNEL: Terminal deoxynucleotidyl transferase dUTP nick end labeling
UHPLC-QTOF-MS: Ultra-high performance liquid chromatography-quadrupole time-of-flight mass spectrometry
UHPLC-Q-Orbitrap HRMS: Ultra-High Performance Liquid Chromatography-Quadrupole Orbitrap High-Resolution Mass Spectrometry
cDNA: Complementary Deoxyribonucleic Acid
DMSO: Dimethyl Sulfoxide
ECL: Enhanced Chemiluminescence
ANOVA: Analysis of Variance
SEM: Standard Error of the Mean
