# Supplementary material for: Rubiadin, as a key metabolite of the Bushen Huoxue formula, promotes apoptosis of endometrial stromal cells and improves intrauterine adhesions by activating the AMPK/p53/p21 pathway
Source: Front Pharmacol. 2026 Apr 24;17:1732284. doi: 10.3389/fphar.2026.1732284 (PMC13153455; doi:10.3389/fphar.2026.1732284)
Supplement: Supplementary file 1 [file Supplementaryfile1.pdf]

Supplement Table S1：

Name	Item number	manufacturer
Rubiadin (purity ≥98%)	CAS No. : 117-02-2	MCE
Dorsomorphin	CAS No. : 866405-64-3	MCE
CCK8 kit	C0037	Beyotime
JC-1 mitochondrial membrane potential detection kit	G1515-100T	Servicebio
Annexin V-FITC/PI flow cytometry apoptosis detection kit	C1383L	Beyotime
anti Rat AMPK	66536-1-Ig	Proteintech
anti Rabbit p-AMPK	AF5908	Beyotime
anti Rabbit p21	10355-1-AP	Proteintech
anti Rabbit Caspase-3	GB11767C	Servicebio
anti Rabbit cleaved Caspase-3	AC033-1	Beyotime
anti Rabbit Bcl-2	GB113375	Servicebio
anti Rabbit Bax	ET1604-34	HUABIO
anti Rabbit COL1A1	AF1840	Beyotime
anti Rabbit β-actin monoclonal antibodies	81115-1-RR	Proteintech
HRP-labeled goat anti-rabbit IgG secondary antibody	RGARO01	Proteintech
Alexa Fluor 488 labeled goat anti-rabbit IgG(H+L)	A0423	Beyotime
TRIzol™ reagent	**15596026CN**	Thermofisher
2×Universal Blue SYBR Green qPCR Master Mix	G3326-15	
RIPA lysis buffer	G2002-100ML	Servicebio
BCA protein quantification kit	P0009	Beyotime
**PAGE Gel Rapid Preparation Kit**	PG112	epizyme
PVDF membrane	IPVH00010	Merck
Fetal bovine serum,	C04001-500	vivacell
DMEM/F-12	C11320033	gibco
Penicillin-Streptomycin-Amphotericin B Additive (100X)	G4015	Servicebio
Lipofectamine 3000	TL301-01/02	Vazyme
Lactate Dehydrogenase Cytotoxicity Assay Kit	C0016	Beyotime
MMP9	CSB-RA014679MA1HU	CUSABIO
TIMP-1	AF8163	Beyotime
Hydroxyproline (HYP) Content Detection Kit	BC0250	Solarbio

Human BCL2 qPCR Primer Pair	QH01409S	Beyotime

Supplement Table S2：

	5’-3’
sh-AMPK	GCATAATAAGTCACAGCCAAA
shNC	TTCTCCGAACGTGTCACGT

Supplement Table S3：

qPCR Primer	Forward primer	Reverse primer
AMPK	5'-ATGGCGCGACCCGGGCTTCTTCT-3'	3'-TACCGCGCTGGGCCCGAAGAAGA-5'
P53	5'-ATGGAGGAGCCGCAGTCAG-3'	3'-TCAGTCTGAGTCAGGCCCTTC-5'
P21	5’-GAGGGCAAGTACGAGTGGCAA-3’	5’-CTGCGCATTGCTCCGCTAACC-3’
Caspase-3	5’-GAGAATTCATGGACGGGTCCGGGGAG-3′	5’-GTCTCGAGCCTCAGCCCATCTTCTTC-3’

Figure S1:

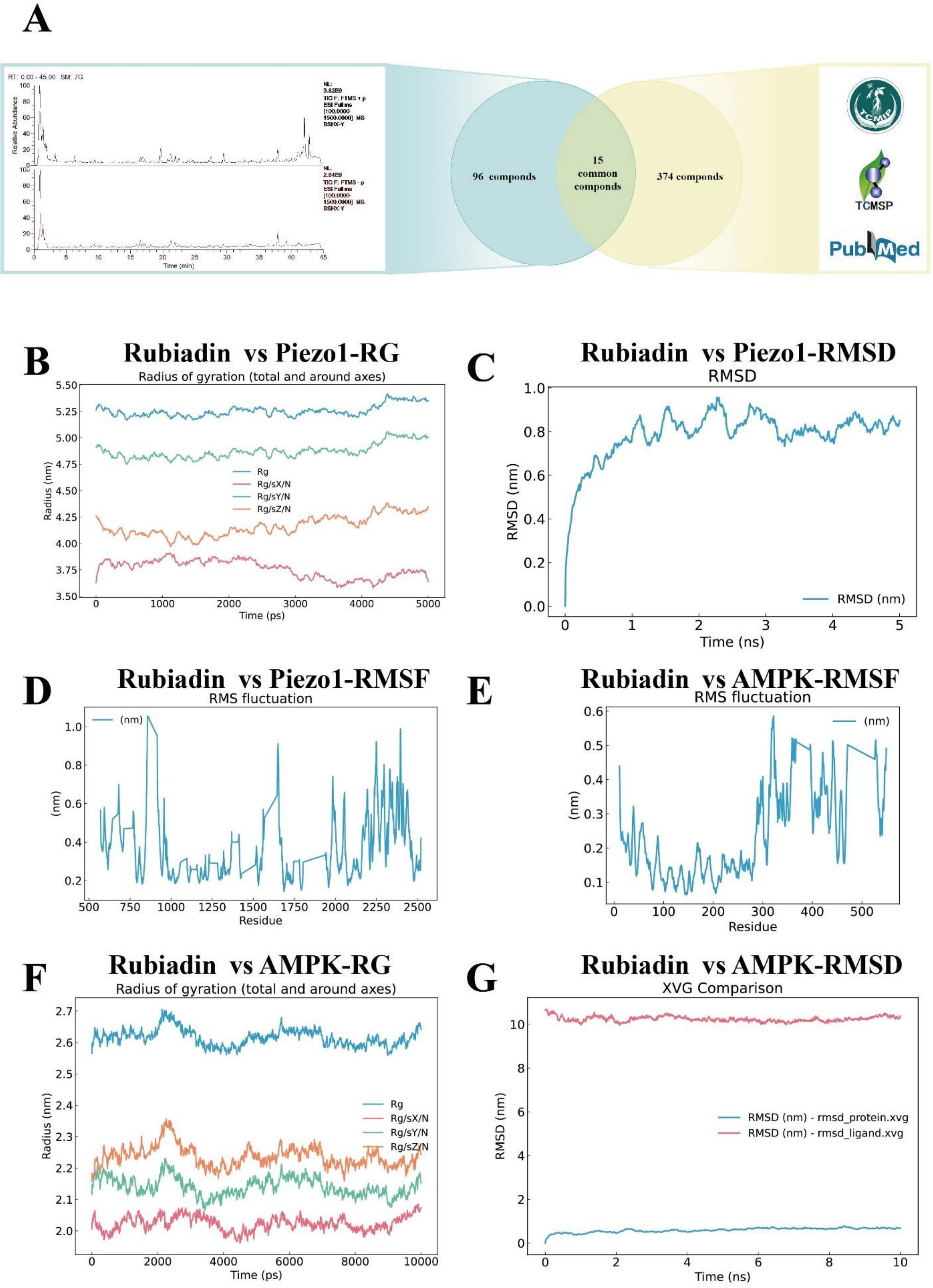

A
96 componds
15 common componds
374 componds
TCMSP
PubMed
B
Rubiadin vs Piezo1-RG
Radius of gyration (total and around axes)
Radius (nm)
Time (ps)
Rg
Rg/sX/N
Rg/sY/N
Rg/sZ/N
C
Rubiadin vs Piezo1-RMSD
RMSD
RMSD (nm)
Time (ns)
D
Rubiadin vs Piezo1-RMSF
RMS fluctuation
(nm)
Residue
E
Rubiadin vs AMPK-RMSF
RMS fluctuation
(nm)
Residue
F
Rubiadin vs AMPK-RG
Radius of gyration (total and around axes)
Radius (nm)
Time (ps)
Rg
Rg/sX/N
Rg/sY/N
Rg/sZ/N
G
Rubiadin vs AMPK-RMSD
XVG Comparison
RMSD (nm)
Time (ns)
RMSD (nm) - rmsd_protein.xvg
RMSD (nm) - rmsd_ligand.xvg

A. Characterization of BSHX metabolites. Left: Total ion chromatogram of BSHX extract. Right: Venn diagram showing the overlap between 96 experimentally identified metabolites and 374 database-predicted compounds, yielding 15 core components.
B. Analysis of the radius of gyration (RG) of the Rubiadin-Piezo1 complex.
C. Root mean square deviation (RMSD) analysis of the Rubiadin and Piezo1 complex.
D. Root mean square fluctuation (RMSF) analysis of the Rubiadin and Piezo1 complex.
Root mean square fluctuation (RMSF) analysis of the E. Rubiadin and AMPK complex.
F. Analysis of the radius of gyration (RG) of the Rubiadin-AMPK complex.
G. Root mean square deviation (RMSD) analysis of the Rubiadin and AMPK complex.

Figure S2:

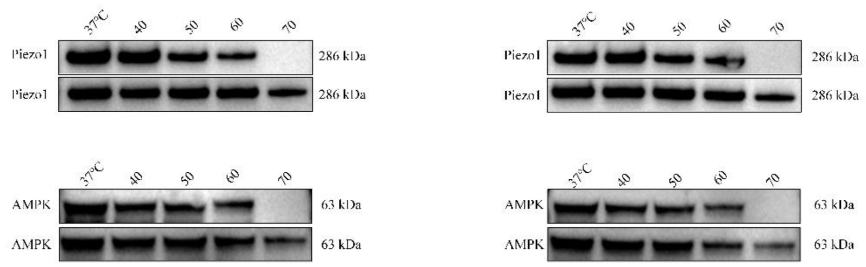

Western blot analysis of Piezo1 and AMPK protein levels under different temperature conditions (Vehicle vs. Compound treatment). Sample size (n = 3) biological replicates; statistical analysis: unpaired two-tailed t-test; data are presented as mean ± SD

Figure S3:

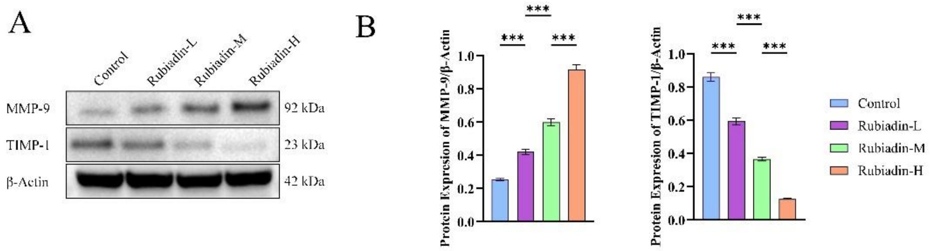

A. Western blot analysis of MMP, TIMP-1 protein levels. Sample size (n = 3) biological replicates;

B. statistical analysis: unpaired two-tailed t-test; data are presented as mean ± SD

Figure S4:

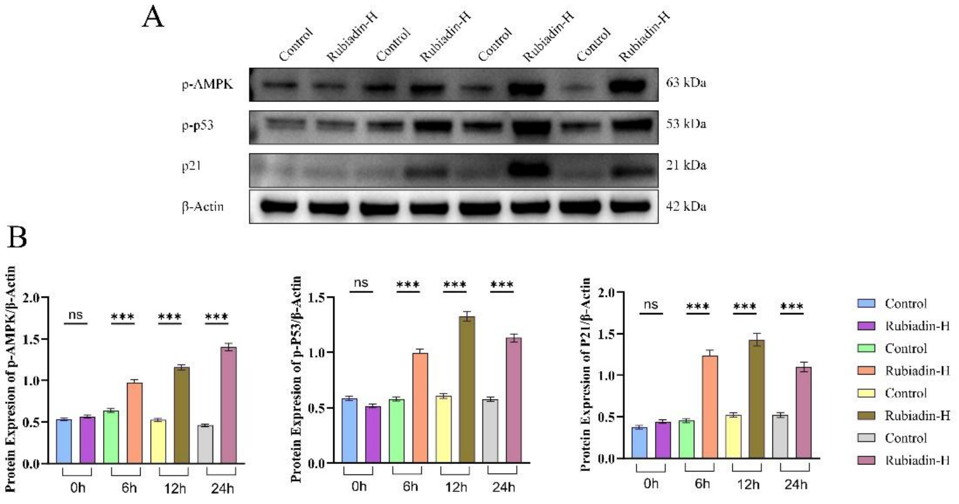

A. Western blot analysis of p-AMPK, p-p53, p21 protein levels. Sample size (n = 3) biological replicates;

B. statistical analysis: unpaired two-tailed t-test; data are presented as mean ± SD.

Figure S5：

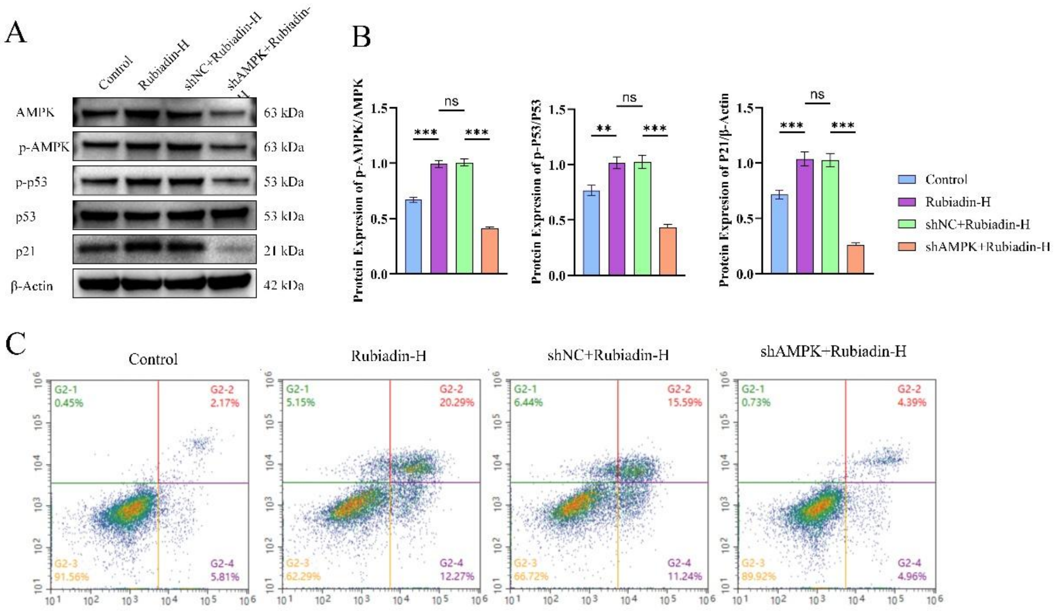

A. Western blot analysis of AMPK, p-AMPK, p53, p-p53, p21 protein levels. Sample size (n = 3) biological replicates;

B. statistical analysis: unpaired two-tailed t-test; data are presented as mean ± SD.

C. Flow cytometry analysis of endometrial stromal cell apoptosis (Annexin V-FITC/PI staining). Sample size (n = 3) cell experimental replicates; statistical analysis: one-way ANOVA + Tukey's post hoc test; data presented as mean ± SEM.
