# Supplementary material for: Rubiadin, as a key metabolite of the Bushen Huoxue formula, promotes apoptosis of endometrial stromal cells and improves intrauterine adhesions by activating the AMPK/p53/p21 pathway
Source: Front Pharmacol. 2026 Apr 24;17:1732284. doi: 10.3389/fphar.2026.1732284 (PMC13153455; doi:10.3389/fphar.2026.1732284)
Supplement: Supplementary file 2 [file Supplementaryfile2.pdf]

Supplement Table S4: Dentification table of chemical components of BSHX

Compond name	TR/min	Mode	Theoretical value m/z	Chemical formula	Area under the blood concentration curve *105
Quinic acid	0.90	[M-H]-	191.0550	C7H12O6	28.86
Gallic acid	1.35	[M+H]+	171.0288	C7H6O5	0.75
Dihydrocinnamol Succinic acid	1.36	[M+HCOO]-	409.1353	C15H24O10	0.92
Crystal lycorin glycoside	1.59	[M-H]-	117.0182	C4H6O4	1313.45
High vanillin acid	1.66	[M-H]-	389.1078	C16H22O11	122.30
Zizulinol	1.68	[M+H]+	183.0653	C9H10O4	20.47
Chlorinated zizulinol	1.69	[M+HCOO]-	407.1200	C15H22O10	334.19
Deacetylated carthaminic acid	2.97	[M+HCOO]-	443.0969	C15H23ClO10	0.94
Cycloarteryl glycoside	3.80	[M-H]-	389.1078	C16H22O11	97.29
Goldenrain alkaloid	4.28	[M+H]+	407.1548	C17H26O11	0.97
Carthaminic acid	4.28	[M+H]+	191.1179	C11H14N2O	0.11
Cryptochromine	5.78	[M-H]-	431.1184	C18H24O12	1.14
Symplocarpine	6.18	[M-H]-	353.0867	C16H18O9	1.59
Peppermint glycoside	7.04	[M+HCOO]-	393.1410	C15H24O9	61.48
Ligustrazine	8.27	[M-H]-	345.1195	C15H22O9	4.54
P-Hydroxybenzaldehyde	8.99	[M-H]-	121.0284	C7H6O2	16.81
Caffeic acid	9.35	[M-H]-	179.0339	C9H8O4	76.20
Isoferulic acid	9.40	[M+H]+	195.0652	C10H10O4	1.10
8-epi-strychnic acid	9.42	[M-H]-	375.1301	C16H24O10	23.50
Neochlorogenic acid	9.42	[M-H]-	353.0867	C16H18O9	2.45
Vanillin acetate	9.42	[M+HCOO]-	239.0550	C10H10O4	21.72
5-O-feruloyl-quinic acid	9.85	[M-H]-	367.1024	C17H20O9	0.98
Guaiacol	9.93	[M-H]-	123.0441	C7H8O2	42.58
M-hydroxybenzaldehyde	9.93	[M+HCOO]-	167.0339	C7H6O2	150.80
Chlorogenic acid	10.32	[M-H]-	353.0867	C16H18O9	2.67
Dihuangneterpene	10.76	[M-H]-	445.2085	C21H34O10	0.14

E/ dihuangneterpene F					
P-hydroxycinnamic acid	12.56	[M-H]-	163.0390	C9H8O3	1077.44
1-acetophenone	12.56	[M-H]-	119.0489	C8H8O	803.30
Geniposide methyl ester	13.14	[M+HCOO]-	433.1357	C17H24O10	1.56
3-O-feruloyl-quinic acid	13.54	[M-H]-	367.1024	C17H20O9	1.45
Atrinoside/dihydro verbenoside	13.76	[M+HCOO]-	435.1514	C17H26O10	0.24
Atrinoside/dihydro verbenoside	14.30	[M+HCOO]-	435.1514	C17H26O10	1.60
Ferulic acid	14.57	[M+H]+	195.0652	C10H10O4	90.16
Ligusticolide I	14.64	[M+H]+	225.1121	C12H16O4	12.79
Hydroxygeniposide	14.91	[M-H]-	403.1251	C17H24O11	0.73
Ligusticolide I isomer	15.69	[M+H]+	225.1121	C12H16O4	2.56
New liquiritin	16.43	[M-H]-	417.1180	C21H22O9	116.12
Chalcone naringenin-4-O-glucoside	16.54	[M-H]-	433.1129	C21H22O10	0.31
Ligusticolide G	16.77	[M+H]+	209.1172	C12H16O3	40.04
Calycosin glucoside	16.89	[M+HCOO]-	491.1184	C22H22O10	10.86
Celiose-isoglycyrrhizin	17.18	[M-H]-	549.1603	C26H30O13	22.73
Apigenin and glycyrrhizin	17.30	[M-H]-	549.1603	C26H30O13	10.81
Hyperoside	18.16	[M-H]-	463.0871	C21H20O12	0.18
Isochlorogenic acid A	18.63	[M-H]-	515.1184	C25H24O12	0.37
Isoquercitrin	18.67	[M-H]-	463.0871	C21H20O12	1.30
morindolide	19.21	[M-H]-	167.0703	C9H12O3	3.97
Rehmannialoside A/B	19.28	[M+HCOO]-	435.2239	C19H34O8	9.68
Ligusticolide H	19.66	[M+H]+	225.1121	C12H16O4	2.43
Red axoidin	20.00	[M-H]-	299.0550	C16H12O6	0.53
4-hydroxy-3-butylphthalide	20.78	[M+H]+	207.1016	C12H14O3	71.38
Licorice phenol	20.92	[M+H]+	367.1176	C21H18O6	0.29
Astragalin	20.98	[M-H]-	447.0922	C21H20O11	1.46

Isochlorogenic acid C	21.19	[M-H]-	515.1184	C25H24O12	0.20
Isoglycyrrhizin	21.30	[M-H]-	255.0652	C15H12O4	49.10
Licorice ketone	21.43	[M+H]+	383.1489	C22H22O6	0.12
Neisoglycyrrhizin	21.84	[M-H]-	417.1180	C21H22O9	12.46
Licuraside	21.91	[M-H]-	549.1603	C26H30O13	1.98
Formononetin	21.95	[M+HCOO]-	475.1235	C22H22O9	6.30
Eriodictyol	22.42	[M-H]-	287.0550	C15H12O6	42.28
New sesamin	22.53	[M-H]-	353.1020	C20H18O6	7.94
Meditol pteroidin	22.54	[M+H]+	301.1071	C17H16O5	99.86
Glyocyrrhizin	22.83	[M+H]+	323.1278	C20H18O4	0.40
jiocarotenoside A1	23.32	[M-H]-	429.2133	C21H34O9	0.16
Liquiritin C2	23.86	[M-H]-	725.2076	C36H38O16	0.15
4, 7-dihydroxy-3-butylphthalide	23.90	[M-H]-	221.0808	C12H14O4	95.22
Naringenin	23.93	[M-H]-	271.0601	C15H12O5	10.63
Calycosin	24.35	[M+H]+	285.0758	C16H12O5	7.70
Astragalus pteroside	24.69	[M-H]-	463.1599	C23H28O10	3.37
7,2' -dihydroxy-3 ',4' -dimethoxyisoflavanone	24.71	[M+H]+	303.1227	C17H18O5	19.38
Licorice chalcone	26.10	[M-H]-	269.0808	C16H14O4	0.18
(Z) -butenylphthalide	27.16	[M+H]+	189.0910	C12H12O2	227.98
(6αR,11αR) 3,9-dimethoxy-10-hydroxypterocaroan	27.56	[M+H]+	301.1071	C17H16O5	4.73
Licorice isoflavone A	27.99	[M+H]+	355.1176	C20H18O6	0.65
4,2',4' -trihydroxychalcone	28.15	[M-H]-	255.0652	C15H12O4	11.37
Rubiadin	28.55	[M-H]-	253.0495	C15H10O4	15.23
Formononetin	29.42	[M-H]-	267.0652	C16H12O4	224.53
Glycyrrhizin P2	30.46	[M-H]-	837.3903	C42H62O17	1.69
apigenin	30.79	[M-H]-	269.0444	C15H10O5	12.69
Uralsaponins F	30.82	[M-H]-	895.3958	C44H64O19	0.37
Ligusticolide E	32.01	[M-H]-	203.0703	C12H12O3	12.69
N-butylphthalide	32.75	[M+H]+	191.1067	C12H14O2	40.44
7,2',4' -trihydroxy-	32.84	[M-H]-	299.0550	C16H12O6	5.42

5-methoxy-3-arylcoumarin					
22-Acetoxyl-glycyrrhizin	33.03	[M-H]-	879.4009	C44H64O18	3.50
3',4', 7-trihydroxyflavone	33.75	[M-H]-	269.0444	C15H10O5	4.45
Achyranthoside E dimethyl ester	36.20	[M-H]-	953.4741	C48H74O19	20.63
Light glycyrrhizin	37.23	[M+H]+	325.1434	C20H20O4	1.20
Ligustilide	37.23	[M+H]+	191.1067	C12H14O2	9.27
Glycyrrhizic acid	37.86	[M-H]-	821.3954	C42H62O16	80.58
Niuxi saponin A	38.73	[M-H]-	925.4791	C47H74O18	0.21
Chikusetsusaponin IV	39.24	[M-H]-	925.4791	C47H74O18	9.06
Glycyrrhizin J2	39.24	[M-H]-	823.4111	C42H64O16	1.08
Ginsenoside Ro	39.34	[M-H]-	955.4897	C48H76O19	8.16
Astragaloside VIII	40.07	[M-H]-	911.4999	C47H76O17	16.96
Huangqiyenins B or Brachyosides B	40.43	[M+HCOO]-	697.4158	C36H60O10	28.11
Isoastragaloside II	40.43	[M+HCOO]-	871.4686	C43H70O15	163.83
Agroastragalosides	41.05	[M+Na]+	833.4658	C43H70O14	16.06

Supplement Table S5： Analysis results of 15 potential active compounds by UHPLC-Q-Orbitrap HRMS

Compond name	TR/min	Mode	Theoretical value m/z	Chemical formula	Area under the blood concentration curve *105
Astragaloside VIII	40.07	[M-H]-	911.4999	C47H76O17	16.96
Formononetin	29.42	[M-H]-	267.0652	C16H12O4	224.53
Glycyrrhizic Acid	37.86	[M-H]-	821.3954	C42H62O16	80.58
Guaiacol	9.93	[M-H]-	123.0441	C7H8O2	42.58
Isoastragaloside II	40.43	[M+HCOO]-	871.4686	C43H70O15	163.83
Isoliquiritigenin	21.3	[M-H]-	255.0652	C15H12O4	49.1
Licoricone	21.43	[M+H]+	383.1489	C22H22O6	0.12
Ligustilide	37.23	[M+H]+	191.1067	C12H14O2	9.27
Medicarpin	22.54	[M+H]+	301.1071	C17H16O5	99.86
Monotropein	1.66	[M-H]-	389.1078	C16H22O11	122.3
Neoisoliquiritin	21.84	[M-H]-	417.118	C21H22O9	12.46
Neoliquiritin	16.43	[M-H]-	417.118	C21H22O9	116.12

Rubiadin	28.55	[M-H]-	253.0495	C15H10O4	15.23
Senkyunolide G	16.77	[M+H]+	209.1172	C12H16O3	40.04
Senkyunolide H	19.66	[M+H]+	225.1121	C12H16O4	2.43

Figure S6:

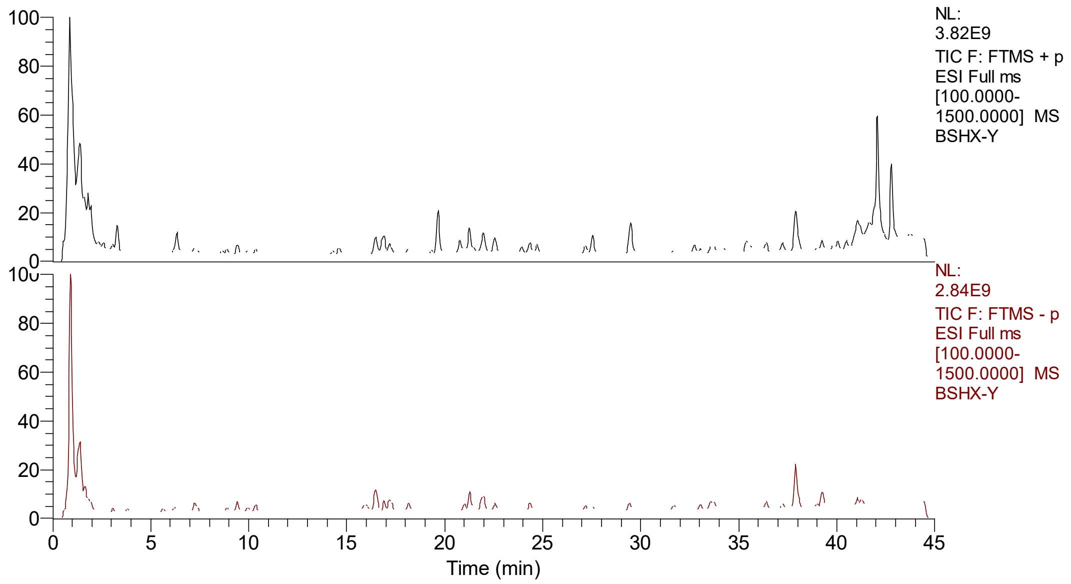

Test sample of the Bushen Huoxue formula decoction - positive and negative total ion current chromatograms

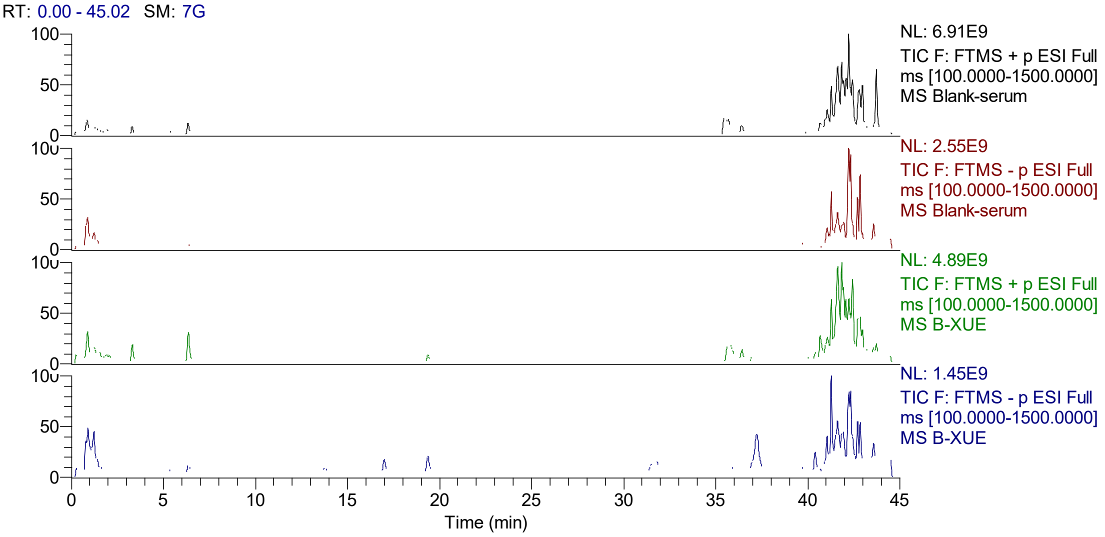

Blank-serum and B-XUE (drug-containing serum)positive and negative ion current diagrams
